# Clear cell renal cell carcinoma: immunological significance of alternative splicing signatures

**DOI:** 10.3389/fonc.2023.1206882

**Published:** 2024-01-15

**Authors:** Jiayu Zhang, Hongyi Jiang, Dapang Rao, Xishi Jin

**Affiliations:** Department of Urology, The Second Affiliated Hospital of Wenzhou Medical University, Wenzhou, China

**Keywords:** clear cell renal carcinoma (ccRCC), alternative splicing (AS), tumor immune microenvironment (TIME), prognosis, immunotherapy

## Abstract

**Background:**

Renal cell carcinoma (RCC) accounts for 90% of renal cancers, of which clear cell carcinoma (ccRCC) is the most usual histological type. The process of alternative splicing (AS) contributes to protein diversity, and the dysregulation of protein diversity may have a great influence on tumorigenesis. We developed a prognostic signature and comprehensively analyzed the role of tumor immune microenvironment (TIME) and immune checkpoint blocking (ICB) treatment in ccRCC.

**Methods:**

To identify prognosis-related AS events, univariate Cox regression was used and functional annotation was performed using gene set enrichment analysis (GSEA). In this study, prognostic signatures were developed based on multivariate Cox, univariate Cox, and LASSO regression models. Moreover, to assess the prognostic value, the proportional hazards model, Kruskal–Wallis analysis, and ROC curves were used. To obtain a better understanding of TIME in ccRCC, the ESTIMATE R package, single sample gene set enrichment analysis (ssGSEA) algorithm, CIBERSORT method, and the tumor immune estimation resource (TIMER) were applied. The database was searched to verify the expression of *C4OF19* in tumor and normal samples. Regulatory networks for AS-splicing factors (SFs) were visualized using Cytoscape 3.9.1.

**Results:**

There were 9,347 AS cases associated with the survival of ccRCC patients screened. A total of eight AS prognostic signatures were developed with stable prognostic predictive accuracy based on splicing subtypes. In addition, a qualitative prognostic nomogram was developed, and the prognostic prediction showed high effectiveness. In addition, we found that the combined signature was significantly associated with the diversity of TIME and ICB treatment-related genes. *C4ORF19* might become an important prognostic factor for ccRCC. Finally, the AS-SF regulatory network was established to clearly reveal the potential function of SFs.

**Conclusion:**

We found novel and robust indicators (i.e., risk signature, prognostic nomogram, etc.) for the prognostic prediction of ccRCC. A new and reliable prognostic nomogram was established to quantitatively predict the clinical outcome. The AS-SF networks could provide a new way for the study of potential regulatory mechanisms, and the important roles of AS events in the context of TIME and immunotherapy efficiency were exhibited. *C4ORF19* was found to be a vital gene in TIME and ICB treatment.

## 1 Introduction

Over the past decades, the global incidence of renal cell carcinoma (RCC) is increasing (1, 2). Among urinary cancers, the mortality rate of renal cell carcinoma ranks first in the world (2). As the main subtype of renal cell carcinoma, clear cell renal carcinomas (ccRCCs) are among the most malignant tumors in urology, responsible for approximately 90,000 deaths annually (3). Approximately 30% of patients with ccRCC have metastases at the first diagnosis, and 20%–40% have recurrence after tumor resection (4, 5). In traditional clinical work, there are some good prognostic biomarkers developed in RCC. However, these approaches may be unreliable due to heterogeneity within the patients (6). Consequently, there is an urgent need for a new approach to predict clinical results more accurately, so as to provide help in choosing treatment strategies.

In recent years, more and more evidence has emphasized the role of immune response as an essential feature of the occurrence and development of ccRCC and therapeutic outcomes (3). Immunotherapy has attracted great attention because of its encouraging results in a variety of malignant tumors (7). Therefore, the most effective strategies were identifying ccRCC patients with molecular signatures, improving prognostic accuracy, and optimizing immunotherapy based on molecular risk distributions.

Alternative splicing (AS) is defined as the process of producing different mRNA splicing isomers from pre-mRNA by different splicing methods (8). AS events were well known for involving AT, AP, AD, AA, ME, ES, and RI. In post-transcriptional regulation, alternative splicing plays a critical role, and more and more studies indicate that alternative splicing is closely linked to cancer cell invasion and metastasis (3, 9, 10). In addition, we learned that splicing factors had a great influence on the regulation of AS events (11). There was a need to mention that abnormal splicing factors could contribute to oncogenic splicing isoforms (12, 13). Unfortunately, there was a lack of adequate understanding of the relationship between the prognostic signature, immunotherapy, and TIME.

In this study, as a result of an integrated analysis of AS events, we characterized TIME and discovered potential molecular mechanisms involved in tumorigenesis. The AS pattern of the KIRC cohort in TCGA was described, and the correlation between AS events and survival was verified using comprehensive bioinformatic analysis. Afterward, the predictive prognostic signatures based on AS events were built and then proven. Next, to meet the clinical application and promote development, we made an AS-clinicopathologic nomogram which could effectively predict the prognosis and guide clinical work. After that, we comprehensively analyzed the association of the prognostic signature newly established with TIME complexity and immune checkpoint blocking (ICB) treatment outcomes. Furthermore, we found a new key gene—*C4ORF19*, and the underlying role of *C4ORF19* in ccRCC was investigated. In the end, we established the AS-SF regulatory network to clarify the underlying mechanisms of ccRCC occurrence and development. The AS-SF networks could provide a new way for the study of potential regulatory mechanisms.

## 2 Materials and methods

### 2.1 Multiomics data acquisition

**Figure 1:**
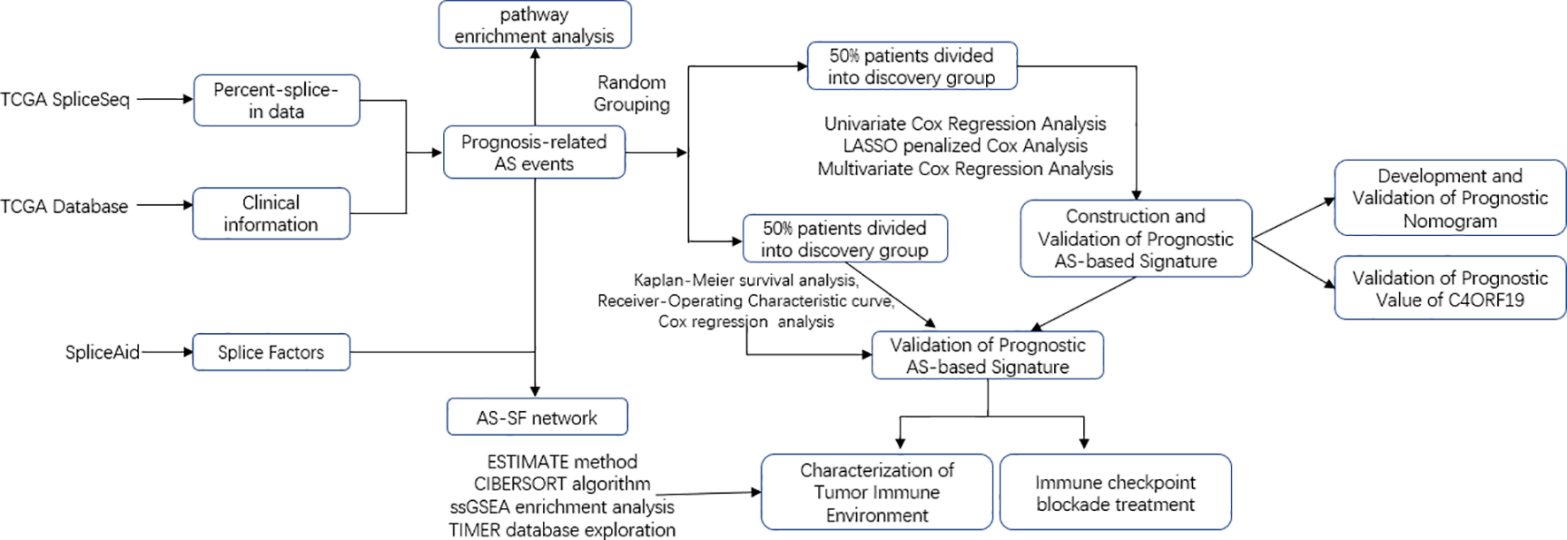
Overall research design. Flow process diagram presenting the process of comprehensive analysis.

The transcriptome and survival data of the ccRCC patients in this study came from The Cancer Genome Atlas portal website (TCGA). Also, the AS data of TCGA came from SpliceSeq, and the SF expression data were obtained from the SpliceAid 2 database (www.introni.it/spliceaid.html). All analyses strictly followed TCGA’s published guidelines, and the detailed analysis flowchart can be found in **Figure 1**.

### 2.2 AS profile recognition process

When setting the PSI value above 0.75 as the point for filtration, samples were partitioned. Using the UpSetR software package, the UpSet plot was drawn and seven subtypes of AS events were found. We named AS by splicing types, ID numbers in splicing sequences, and corresponding parental gene names. There was a case that *C4orf19* was the corresponding parent gene name, 69001 was the ID number in SpliceSeq, and AT was the splicing type in “*C4orf19*|69001|AT”.

### 2.3 Screening AS events associated with survival

When we detected the PSI standard deviation less than 0.01, the data of AS events were deleted. The connection between the overall survival (OS) and AS events was found in the univariate Cox regression analysis (**Additional file 1: Table 1**), which was exhibited in the UpSet map and volcano map. In addition, each bubble chart of the seven subtypes summarized the 20 most important AS events.

### 2.4 Prognostic signature and nomogram

Firstly, candidate models for each splicing pattern were determined by least absolute shrinkage and selection operator (LASSO) regression analysis, in which way we could also avoid model overfitting. Next, multivariate Cox regression analysis was applied to screen prognostic predictors from the identified AS events. Because the pattern of AS events in post-transcriptional modification was independent of each subtype, the AS events identified in each of the splicing subtypes described above were integrated and then another prognostic feature was generated. Afterward, risk scores were calculated according to the formula: risk score = βAS event1 × PSIAS event1 + ⋯ + βAS eventn × PSIAS eventn. The specific formulas for each prognostic signature can be found in **Additional file 1: Table 2**. Consequently, the low-risk group and the high-risk group were born by the calculated median risk scores. The “survival” R package was employed to analyze K–M survival curves. The predictive value of this prognostic signature was validated by using time-dependent receiver operating characteristic (ROC) curves. Then, univariate and multivariate Cox regression analyses were exploited to ascertain whether this signature could be used as an independent prognostic factor. In addition, stratified survival analysis further verified whether prognostic performance in patients was independent of clinical data including age; sex; pathological grade; T, N, and M categories; and tumor stage. Then, we calculate the AUC from the ROC curve to systematically measure the value of the accuracy of the model for 1-, 2-, and 3-year OS. Finally, to accurately calculate the OS of ccRCC patients, we established prognostic nomograms to obtain the survival probabilities of 1, 2, and 3 years. Then, there was a calibration curve showing the prognostic value of the AS-constructed nomogram. It should be noted that the model was highly predictive when the calibration curve was close to 45°.

### 2.5 Risk score and characteristics of tumor-infiltrating immune cells

Information on immune infiltrates such as B cells from each specimen was downloaded from TIMER. The ssGSEA algorithm of the R package “GSEAbase” was performed to elucidate the enrichment of two different risk subgroups in 29 gene sets related to immune function. Subsequently, we calculated the purity of the tumor and the degree of cell invasion (stromal and immune cells) using the R package “ESTIMATE” to validate the significantly different characteristics of the TIME between the low-risk and high-risk groups. The proportion of 22 immune cell types in the tumor sample was recognized by assessing the relative subsections of RNA transcripts from CIBERSORT.

### 2.6 ICB treatment

According to existing research, the expression level of key genes associated with immune checkpoint blockade might have a close relationship with the clinical results of ICB treatment (14, 15). Six key genes (PD‐L1, IDO1, PD‐L2, PD‐1, CTLA‐4, and TIM‐3) of immune checkpoint blockade therapy in ccRCC (16, 17) were obtained. Afterward, to investigate the potential role of risk score in immune checkpoint blockade therapy of ccRCC, AS-based prognostic characteristics were significantly related to the expression levels of four key genes for immune checkpoint blockade. At last, the expression levels of 47 immune checkpoint genes (i.e., CTLA4, BTLA, etc.) were compared in low-risk and high-risk patients.

### 2.7 Splicing regulatory network

A total of 404 SFs derived from a previous study (18) are exhibited in **Additional file 1: Table 3**, and the RNA-seq profiles of SFs can be found in the TCGA database. In addition, we conducted a Spearman correlation analysis to assess the connection between SFs and survival-related AS events. *p <*0.001 and correlation coefficient >0.6 were the cutoff values. In the end, Cytoscape (version 3.9.1) was applied to build an underlying SF-AS regulatory network.

### 2.8 Experimental proof

#### 2.8.1 Immunohistochemistry

From Outdo Biotech (Shanghai, China), we purchased one ccRCC tissue microarray (TMA, Cat. HKid-CRCC060PG-01). TMA HKID-CRCC060PG-01 contained 30 paired adjacent tissues and 30 ccRCC tissues. Moreover, Outdo Biotech (Shanghai, China) also provided detailed clinicopathological features of this TMA, and TMA was approved ethically by the Clinical Research Ethics Committee, Outdo Biotech (Shanghai, China).

On TMA samples of ccRCC tissues, immunohistochemistry (IHC) was performed according to the standard procedure. For antigen retrieval, EDTA was used, and the primary antibodies were incubated overnight at 4°C. The primary antibody used in the study was anti-*C4ORF19* (1:500 dilution; Cat. PA5-60368, RRIDP: AB_2639064, Thermo Fisher Scientific). Lastly, using Aperio Digital Pathology Slide Scanners, stained TMA was scanned to visualize antibody staining and hematoxylin counterstaining.

#### 2.8.2 Real-time polymerase chain reaction

Human renal cancer tissue and adjacent/normal tissue came from the biological sample library of the Second Affiliated Hospital of Wenzhou Medical University (Yuying Children’s Hospital of Wenzhou Medical University). The sample numbers were KI220001, KI220002, KI220003, LI220005, and KI220006. Quantitative real-time polymerase chain reaction (qRT-PCR) was performed (approval nos. 2022-K-151-01, 2022-K-151-02, and 2022-K-151-03 by the ethics committee).

According to the extraction standards provided by the reagent manufacturer, TRIzol kit (Invitrogen, Carlsbad, CA, USA) was used to extract total RNA (tRNA). We used a NanoDrop 2000 spectrophotometer to determine RNA concentration and purity. In the following steps, total RNA was reverse-transcribed into cDNA using the RevertAid First Strand cDNA Synthesis Kit (TaKaRa:Tokyo, Japan). SYBR Green detection reagent (TaKaRa) and LightCycler^®^ 96 Real-Time PCR System (Roche, IN, USA) were used for quantitative polymerase chain reaction (qPCR). Finally, the 2^−ΔΔCq^ method was used to examine gene expression data. All primers were synthesized by Sangon Biotech (Shanghai, China). The sequences of all primers used in qPCR are shown in **Table 1**.

**Table 1:** Sequences of all primers used in qPCR.

Genes	Forward primer sequence (5′–3′)	Reverse primer sequence (5′–3′)
*C4orf19*	CAGCCTGGGTGACAGTGCAA	AACCAGCTCGGTCCCTTCCT
*GADPH*	GCGGGGCTCTCCAGAACATC	TCCACCACTGACACGTTGGC

### 2.9 Statistical analysis

In this study, for comparisons between two different groups, we used the Wilcoxon test, and for comparisons between more than two groups, we used the Kruskal–Wallis test. OS was the time between diagnosis and death. The K–M log-rank test was employed to plot the survival curse. Moreover, the Pearson correlation test was applied to explore the correlation between risk score, clinical variables, and degree of immune cell infiltration and immune checkpoint. When the result of the CIBERSORT algorithm *p* ≥ 0.05, further study was abandoned. Then, in order to verify the independent prognostic prediction abilities of risk signatures, univariate and multivariate analyses were carried out by the Cox regression model. For 1-, 2-, and 3-year OS, we used ROC curves to evaluate their prognostic value. *p <*0.05 was regarded as statistically significant. All statistical analyses were performed using R software in version 4.1.2.

## 3 Results

### 3.1 Basic information on patients and AS events in ccRCC

**Figure 2:**
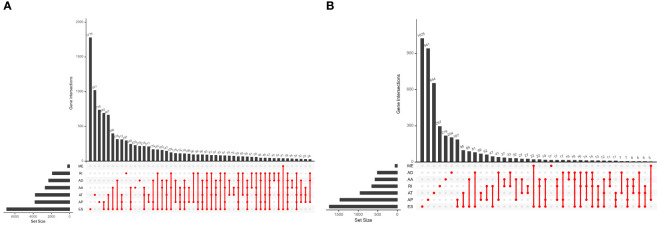
**(A)** The UpSet plot of gene interactions among the seven types of AS events in the TCGA KIRC cohort. **(B)** The UpSet plot of gene interactions among the seven types of prognostic relevant AS events.

Five hundred thirty-seven patients with ccRCC were obtained from the TCGA database, and 11 patients without complete information were rejected. Therefore, 526 patients in total were included. **Table 2** presents the basic clinical data of all ccRCC patients. In addition, by using the UpSet plot (**Figure 2A**), we analyzed the AS event profiles comprehensively and displayed gene intersections among the seven subtypes of AS events. It could be seen that ES was the most frequent splicing pattern, while ME was the least frequent.

**Table 2:** Baseline data of all ccRCC patients.

Characteristics	Type	*N*	Proportion
Age	≤65	352	65.55%
	>65	185	34.45%
Gender	Female	191	35.57%
	Male	346	64.43%
Grade	G1–2	244	45.44%
	G3–4	285	53.07%
	Unknown	8	1.49%
Stage	I–II	326	60.71%
	III–IV	208	38.73%
	Unknown	3	0.56%
T stage	T1–2	344	64.06%
	T3–4	193	35.94%
M stage	M0	426	79.33%
	M1	79	14.71%
	Unknown	32	5.96%
N stage	N0	240	44.69%
	N1	17	3.17%
	Unknown	280	52.14%

### 3.2 Finding survival-related AS events

**Figure 3:**
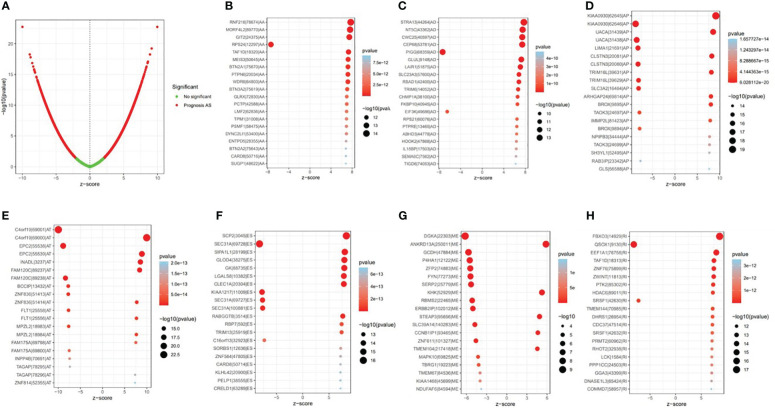
The survival-relevant alternative splicing (AS) events. **(A)** The volcano plots of survival-relevant AS events. The most significant survival-relevant AAs, ADs, APs, ATs, ESs, MEs, and RIs in the TCGA KIRC cohort **(B–H)**.

Univariate Cox regression analysis showed that 9,347 AS events were significantly associated with survival (*p* < 0.05). Furthermore, a detailed record of the data can be found in **Additional file 1: Table 1**. In **Figure 2B**, the gene interactions among the seven types of survival-related AS events are shown. Moreover, ES was still the main splicing pattern. On the other hand, the volcano map was designed to present the distribution of AS events (**Figure 3A**), and the top 20 AS events with significant survival correlation from seven subtypes were summarized by using the bubble graphs (**Figures 3B–H**).

### 3.3 Establishment of the verified prognostic signature

**Figure 4:**
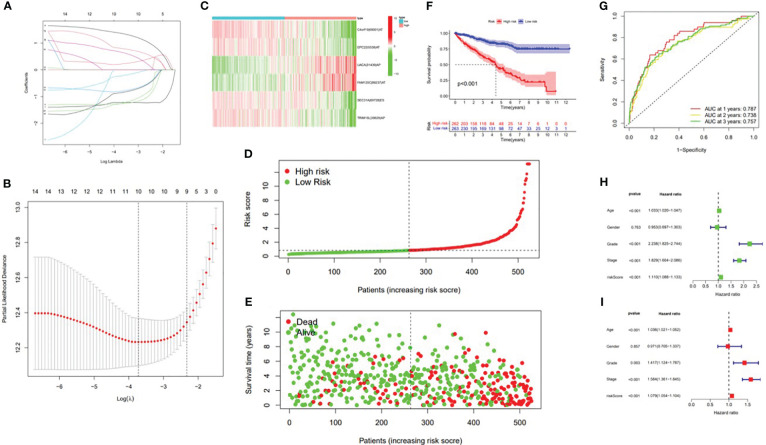
Confirmation of the ALL AS-based prognostic signature. **(A)** Least absolute shrinkage and selection operator (LASSO) coefficient profiles of the whole AS events. **(B)** Ten times cross‐validation for tuning parameter selection in the LASSO regression. **(C)** Heatmap of the percent spliced index (PSI) value of ALL signature AS events in clear cell renal carcinoma (ccRCC). The colors from red to green show a trend from high expression to low expression. **(D)** Distribution of the ALL signature risk score. **(E)** The survival status and duration of ccRCC patients. **(F)** The K–M curve presenting survival in the high-risk and low-risk sets. **(G)** ROC analysis of the risk scores for overall survival prediction. The AUC was calculated for ROC curves, and sensitivity and specificity were calculated to assess score performance. Proportional hazards model results. **(H)** Univariate Cox regression results. **(I)** Multivariate Cox regression results.

**Figure 5:**
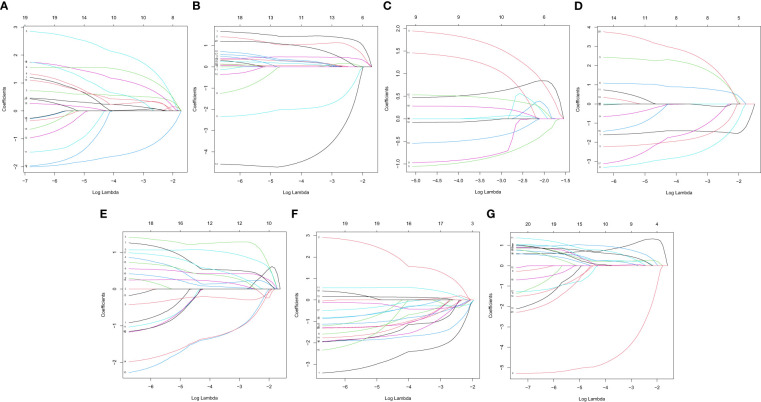
LASSO coefficient of prognostic relevant AS events. **(A)** AA. **(B)** AD. **(C)** AP. **(D)** AT. **(E)** ES. **(F)** ME. **(G)** RI.

**Figure 6:**
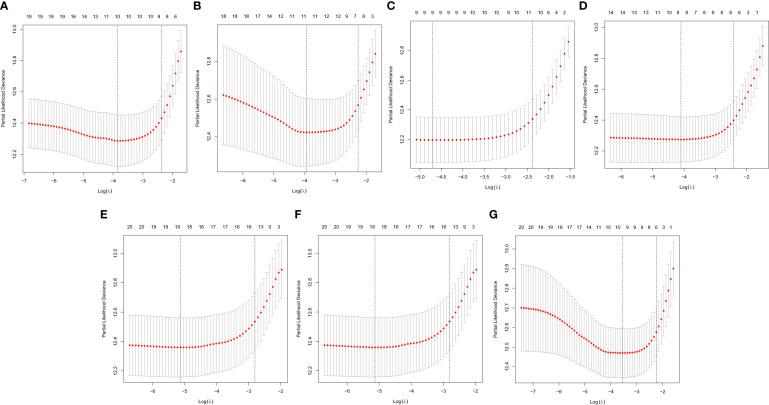
A graph of the error rate of cross-validation. **(A)** AA. **(B)** AD. **(C)** AP. **(D)** AT. **(E)** ES. **(F)** ME. **(G)** RI.

**Figure 7:**
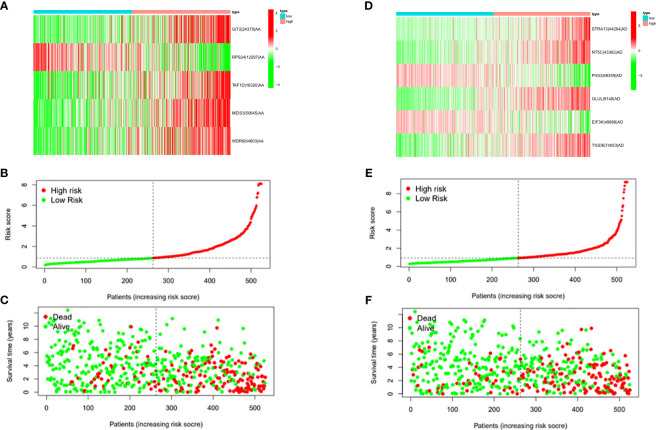
**(A)** Heatmap of the PSI value of AA events in ccRCC. The colors from red to green show a trend from high expression to low expression. **(B)** Distribution of the AA prognostic signature risk score. **(C)** The survival status and duration of ccRCC patients in the AA prognostic signature. **(D)** Heatmap of the PSI value of AD events in ccRCC. The colors from red to green show a trend from high expression to low expression. **(E)** Distribution of the AD prognostic signature risk score. **(F)** The survival status and duration of ccRCC patients in the AD prognostic signature.

**Figure 8:**
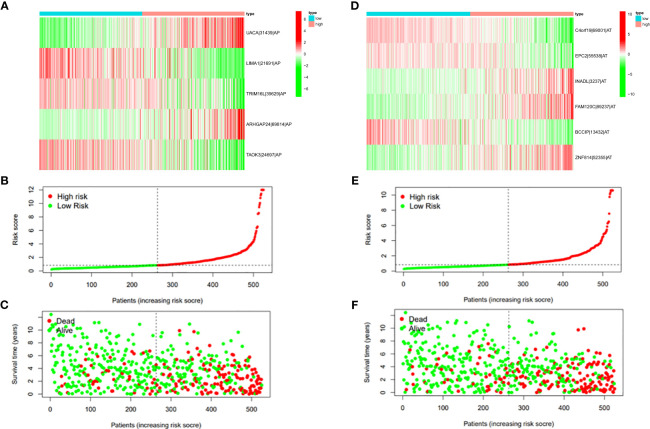
**(A)** Heatmap of the PSI value of AP events in ccRCC. The colors from red to green show a trend from high expression to low expression. **(B)** Distribution of the AP prognostic signature risk score. **(C)** The survival status and duration of ccRCC patients in the AP prognostic signature. **(D)** Heatmap of the PSI value of AT events in ccRCC. The colors from red to green show a trend from high expression to low expression. **(E)** Distribution of the AT prognostic signature risk score. **(F)** The survival status and duration of ccRCC patients in the AT prognostic signature.

**Figure 9:**
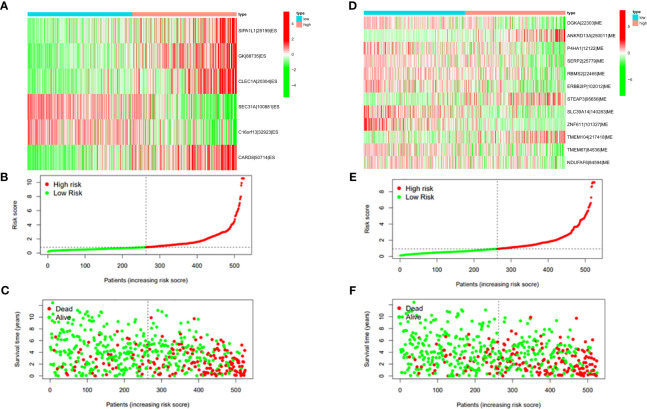
**(A)** Heatmap of the PSI value of ES events in ccRCC. The colors from red to green show a trend from high expression to low expression. **(B)** Distribution of the ES prognostic signature risk score. **(C)** The survival status and duration of ccRCC patients in the ES prognostic signature. **(D)** Heatmap of the PSI value of ME events in ccRCC. The colors from red to green show a trend from high expression to low expression. **(E)** Distribution of the ME prognostic signature risk score. **(F)** The survival status and duration of ccRCC patients in the ME prognostic signature.

**Figure 10:**
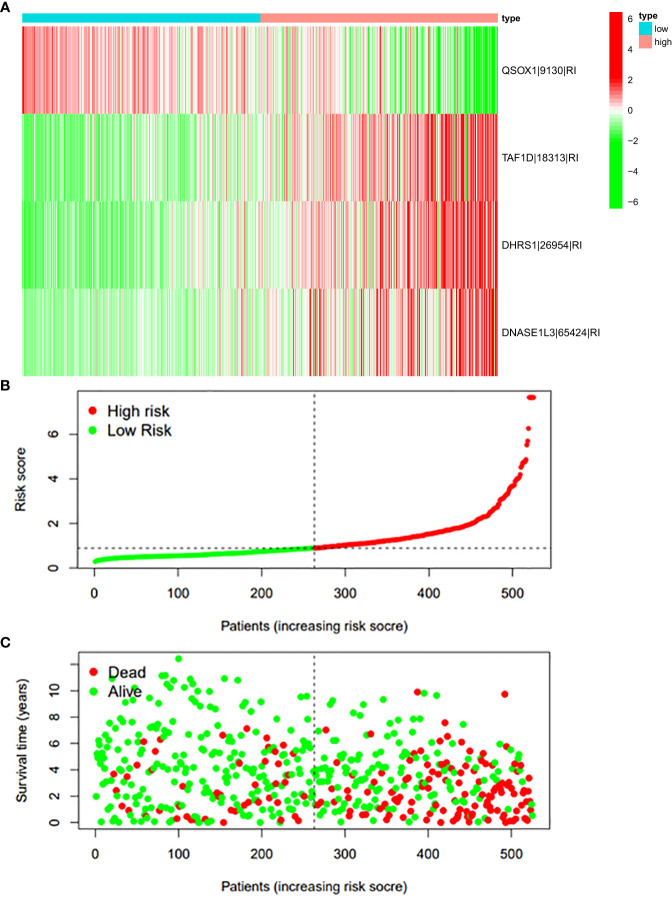
**(A)** Heatmap of the PSI value of RI events in ccRCC. The colors from red to green show a trend from high expression to low expression. **(B)** Distribution of the RI prognostic signature risk score. **(C)** The survival status and duration of ccRCC patients in the RI prognostic signature.

**Figure 11:**
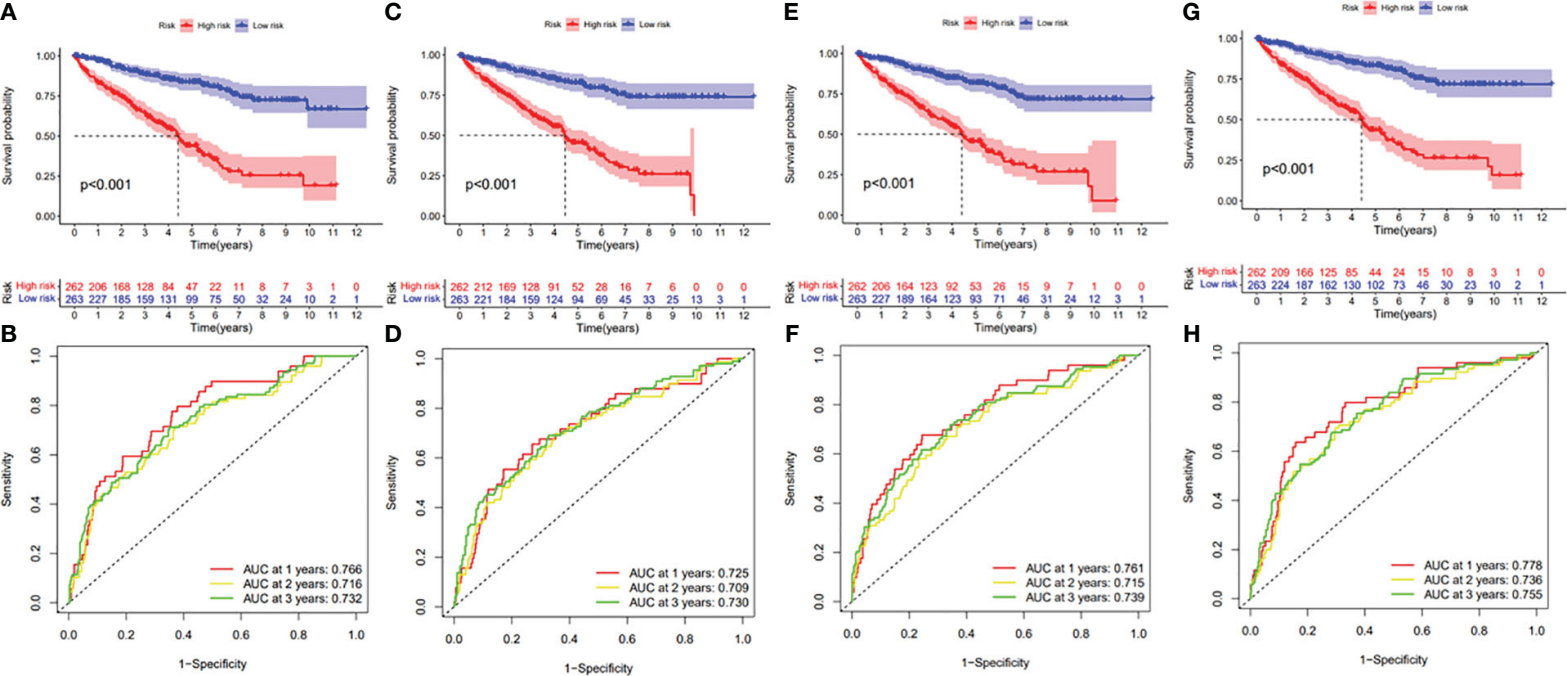
**(A)** Kaplan–Meier curve presenting survival in the AA prognostic signature. **(B)** ROC analysis of the risk scores in the AA prognostic signature. **(C)** Kaplan–Meier curve presenting survival in the AD prognostic signature. **(D)** ROC analysis of the risk scores in the AD prognostic signature. **(E)** Kaplan–Meier curve presenting survival in the AP prognostic signature. **(F)** ROC analysis of the risk scores in the AP prognostic signature. **(G)** Kaplan–Meier curve presenting survival in the AT prognostic signature. **(H)** ROC analysis of the risk scores in the AT prognostic signature.

**Figure 12:**
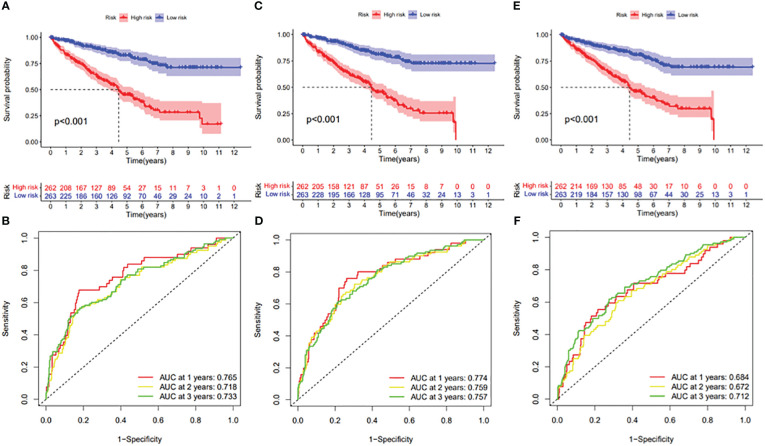
**(A)** Kaplan–Meier curve presenting survival in the ES prognostic signature. **(B)** ROC analysis of the risk scores in the ES prognostic signature. **(C)** Kaplan–Meier curve presenting survival in the ME prognostic signature. **(D)** ROC analysis of the risk scores in the ME prognostic signature. **(E)** Kaplan–Meier curve presenting survival in the RI prognostic signature. **(F)** ROC analysis of the risk scores in the RI prognostic signature.

**Figure 13:**
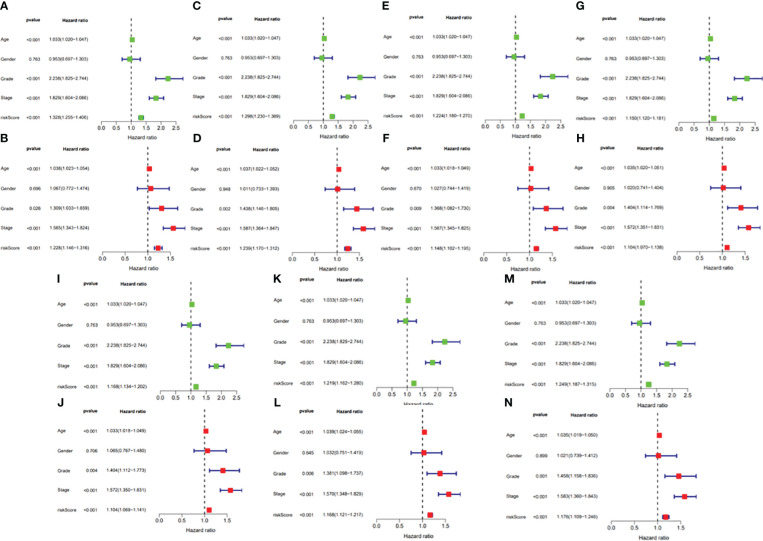
**(A)** Univariate Cox regression analyses in the AA prognostic signature. **(B)** Multivariate Cox regression analyses in the AA prognostic signature. **(C)** Univariate Cox regression analyses in the AD prognostic signature. **(D)** Multivariate Cox regression analyses in the AD prognostic signature. **(E)** Univariate Cox regression analyses in the AP prognostic signature. **(F)** Multivariate Cox regression analyses in the AP prognostic signature. **(G)** Univariate Cox regression analyses in the AT prognostic signature. **(H)** Multivariate Cox regression analyses in the AT prognostic signature. **(I)** Univariate Cox regression analyses in the ES prognostic signature. **(J)** Multivariate Cox regression analyses in the ES prognostic signature. **(K)** Univariate Cox regression analyses in the ME prognostic signature. **(L)** Multivariate Cox regression analyses in the ME prognostic signature. **(M)** Univariate Cox regression analyses in the RI prognostic signature. **(N)** Multivariate Cox regression analyses in the RI prognostic signature.

In this study, the prognostic abilities of the survival-related AS events found in the previous step were evaluated by using the stepwise LASSO algorithm and multivariate Cox regression analysis. Moreover, the LASSO regression analysis results of ALL AS events and seven AS event subtypes are exhibited in **Figures 4A, B**, **5A–G**, **6A–G**. Next, the best survival-related AS events, which were determined by multivariate Cox analysis, were performed to build eight AS prognostic signatures, namely, AA, AD, AP, AT, ES, ME, RI, and ALL. The formulas for each prognostic signature are detailed in **Additional file 1: Table 2**. Using the median risk score as a standard for further study, ccRCC patients were ranked into low- and high-risk groups. The distribution of eight different AS events (AA, AP, AT, AD, ME, RI, ES, and ALL) and their PSI values in the two subgroups and patients was exhibited in the heatmap (**Figures 4C**, **7A, D**, **8A, D**, **9A, D**, **10A**). In the same way, the distribution of risk score (**Figures 4D**, **7B, E**, **8B, E**, **9B, E**, **10B**) and the dot plot of survival status (**Figures 4E**, **7C, F**, **8C, F**, **9C, F**, **10C**) indicated a lower overall survival in the higher-risk patients. Furthermore, the Kaplan–Meier curve also confirmed that in the low-risk subgroup, patients had a significantly better prognosis than those in the high-risk subgroup (**Figures 4A**, **11A, C, E, G**, **12A, C, E, G**; all *P* < 0.05). The results showed that the areas under the risk score curves of 1-, 2- and 3-year survival were all greater than 0.70, indicating that the established prognostic signature had highly sensitive and specific survival prediction ability (**Figures 4G**, **11B, D, F, G**, **12B, D, F, G**). Moreover, the risk score might become an independent prognostic signature of the ccRCC (univariate Cox model in **Figures 4H** and **13A, C, E, G, I, K, M** and multivariate Cox regression analysis in **Figures 4I** and **13B, D, F, H, J, L, N**).

### 3.4 Construction of the verified nomogram

**Figure 14:**
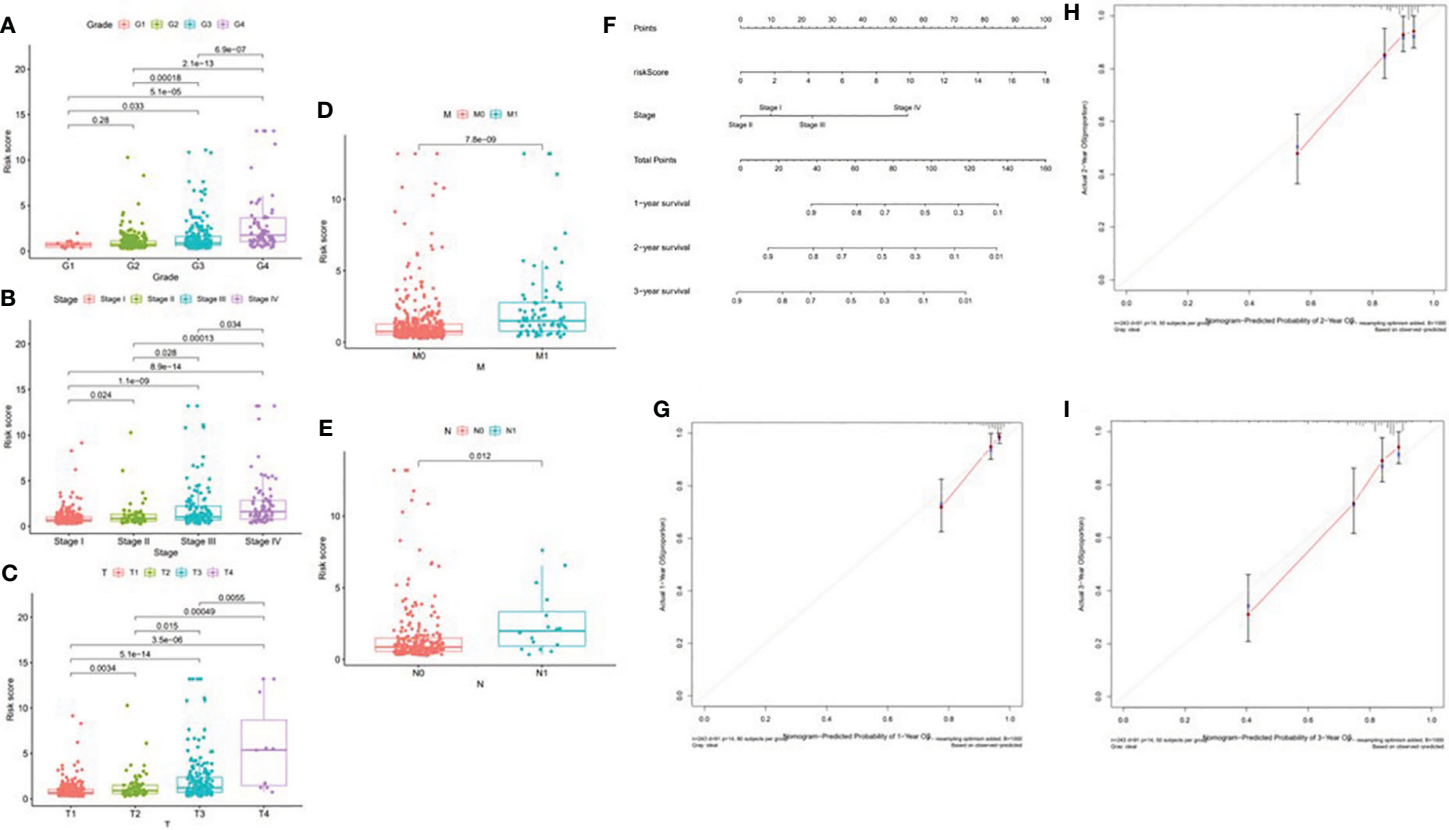
Correlation of risk score with clinical features and construction of nomogram. **(A)** Correlation of risk score with tumor grade. **(B)** Correlation of risk score with clinicopathological stage. **(C)** Correlation of risk score with T status. **(D)** Correlation of risk score with M status. **(E)** Correlation of risk score with N status. **(F)** A nomogram was constructed by stage and risk signature for predicting the survival of ccRCC patients. **(G)** One‐year nomogram calibration curves. **(H)** Two‐year nomogram calibration curves. **(I)** Three‐year nomogram calibration curves.

According to the difference in the risk score in different subtypes of clinical variables, clinical significance was explored. With the progression of tumor grade (most *p* < 0.05, **Figure 14A**); clinicopathological stage (most *p* < 0.05, **Figure 14B**); and T, M, and N stages (most *p* < 0.05, **Figures 14C–E**), the risk score significantly rose, suggesting that prognostic risk score had a positive correlation with tumor progression. Next, the prognostic nomogram established for forecasting the prognosis of ccRCC patients is exhibited in **Figure 14F**. It was well known that there was a great prognostic capability of 1-, 2-, and 3-year OS in the nomogram plot when the calibration curve was close to the diagonal (**Figures 14G–I**).

### 3.5 Risk score and TIME characterization

**Figure 15:**
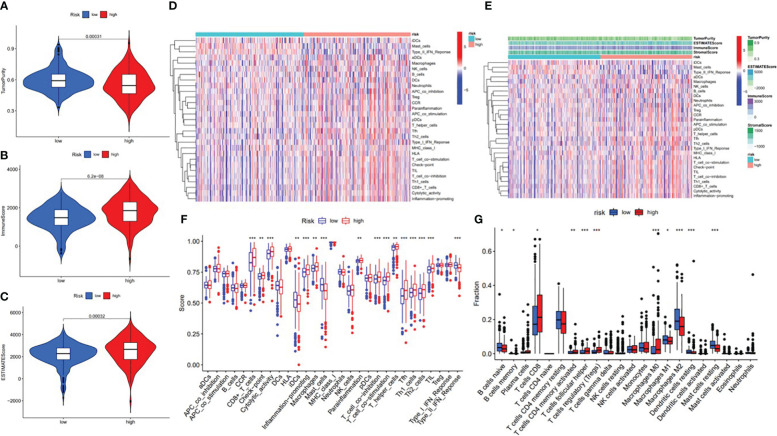
Correlation between infiltrating immune cells and the ALL AS-based prognostic signature. **(A)** Comparison of tumor purity between the low- and high-risk groups. **(B)** Comparison of immune score between the low- and high-risk groups. **(C)** Comparison of ESTIMATE score between the low- and high-risk groups. **(D)** Heatmap exhibited enrichment of 29 immune signatures of the low-/high-risk groups. Blue represents low activity and red represents high activity. **(E)** Heatmap of 29 immune signatures and immune scores of two different risk score groups. Blue represents low activity and red represents high activity. **(F)** Difference of enrichment of immune-related signatures between the low-risk and high-risk groups. **(G)** Distinction of infiltrating immune cell subpopulations and levels between the low-/high-risk groups. * means p<0.05, * * means p<0.01, * * * means p<0.001.

**Figure 16:**
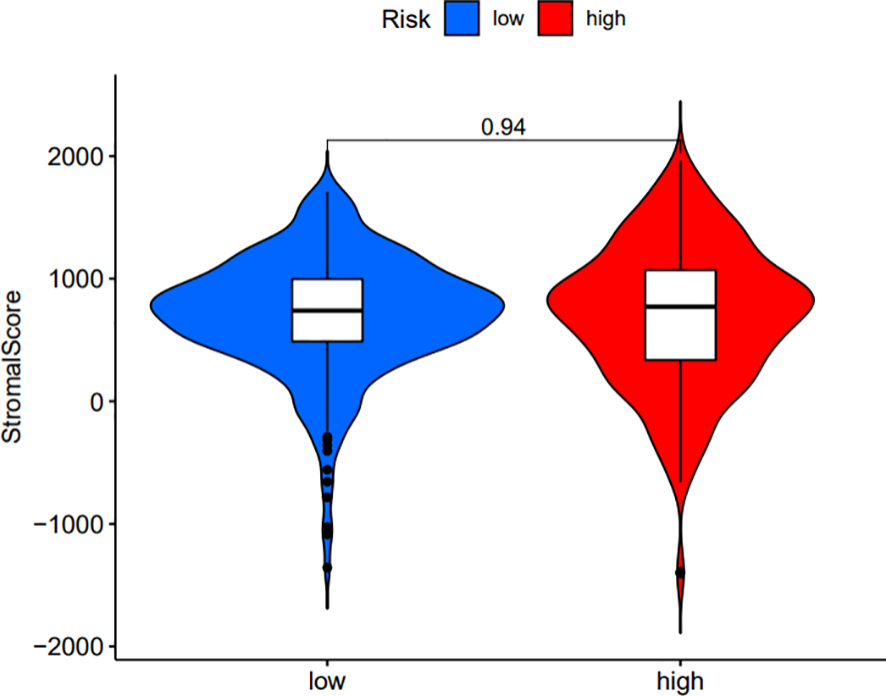
Comparison of the stromal score between the low-/high-risk groups.

In order to further investigate the possibility of using risk score as an immune indicator, we performed correlation analyses between risk score and immune score (from the ESTIMATE algorithm), ssGSEA characteristics, and TIC subtypes and levels (from the CIBERSORT method). The high-risk patients achieved a higher immune score and ESTIMATE score and lower tumor purity (**Figures 15A–C**), which suggested higher immune infiltration. However, there was no significant difference in stromal score (**Figure 16**). Then, in **Figures 15D, E**, immune-related signatures were shown to differ between the two subgroups, where immunological scores corresponding to immune-related signatures were exhibited for each patient in the low-/high-risk group. The results revealed that the infiltration of immune cells such as CD8+ T cells, macrophages, T helper cells, Tfh, Th1 cells, Th2 cells, and TIL and the immune signatures such as parainflammation, T-cell co-inhibition, T-cell co-stimulation, checkpoint, inflammation-promoting, and cytolytic activity were significantly increased with increased risk score (**Figure 15F**). On the contrary, iDCs, mast cells, and type IIIFN response were significantly decreased with increased risk score (**Figure 15F**). The CIBERSORT algorithm results showed that the proportion of CD8+ T cells, activated CD4 memory T cells, follicular helper T cells, Tregs, and M0 macrophages was positively associated with risk score, and the proportion of naive B cells, memory B cells, M1 macrophages, M2 macrophages, resting dendritic cells, and resting mast cells was negatively associated with risk score (**Figure 15G**). In conclusion, the ALL prognostic signature could be a kind of new method to clarify the ccRCC immunoregulatory network.

### 3.6 Correlation between the ALL prognostic signature and ICB key therapy

**Figure 17:**
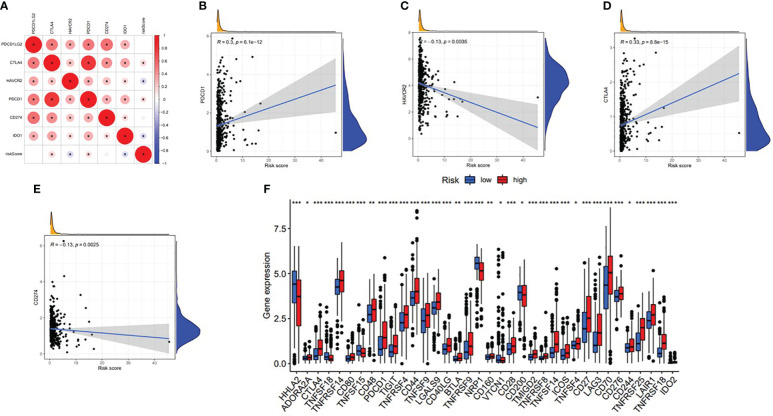
Association between the ALL AS-based prognostic signature and key immune checkpoint genes. **(A)** Correlation analyses between immune checkpoint inhibitors CD274, PDCD1, PDCD1LG2, CTLA4, HAVCR2, and IDO1 and risk score. **(B)** Correlation between risk score and PDCD1. **(C)** Correlation between risk score and HAVCR2. **(D)** Correlation between risk score and CTLA4. **(E)** Correlation between risk score and CD274. **(F)** Comparison of immune checkpoint blockade-related gene expression levels between the low-risk group and high-risk groups. *means p<0.05, * * means p<0.01, * * * means p<0.001.

With the increasing attention paid to ICB therapy in clinical work, immune checkpoint inhibitors have greatly changed the clinical decision-making of cancer oncology (19, 20). We screened out six key immune checkpoint inhibitor genes (PDCD1, CD274, PDCD1LG2, CTLA‐4, HAVCR2, and IDO1) (21, 22) for further analysis. Then, to uncover the potential role of risk signature in ICB therapy for ccRCC, we comprehensively analyzed the association between the ALL prognostic signature and ICB key targets (**Figure 17A**). The results showed that the ALL prognostic signature had a significant positive association with PDCD1 (*r* = 0.3; *p* = 6.1e−12; **Figure 17B**) and CTLA4 (*r* = 0.33; *p* = 8.8e−15; **Figure 17D**) and a significant negative association with HAVCR2 (*r* = −0.13; *p* = 0.0035; **Figure 17C**) and CD274 (*r* = −0.13; *p* = 0.0025; **Figure 17E**). Furthermore, 36 of the 47 (i.e., HHLA2, CD44, etc.) ICB key gene expression levels between the low- and high-risk groups were significantly dysregulated in the further correlation analysis (**Figure 17F**). These results suggested that the level of the ALL prognostic signature does affect the expression changes of ICB key genes, which could be a valuable factor.

### 3.7 Role of *C4ORF19* in the prognosis and ICB treatment of vital genes

**Figure 18:**
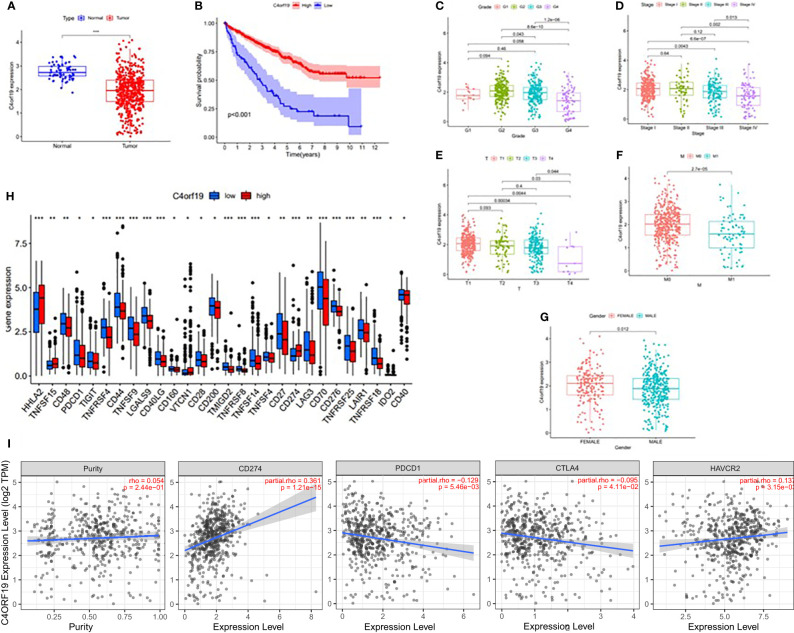
The clinical significance of *C4ORF19* in ccRCC. **(A)** *C4ORF19* was of lower expression in ccRCC tumor tissue than in normal tissue. **(B)** Higher ZDHHC16 expression levels revealed longer overall survival. **(C)** Correlation of *C4ORF19* expression with tumor grade. **(D)** Correlation of *C4ORF19* expression with major pathological stages. **(E)** Correlation of *C4ORF19* expression with T status. **(F)** Correlation of *C4ORF19* expression with M status. **(G)** Correlation of *C4ORF19* expression with gender. **(H)** Comparison of immune checkpoint blockade-related gene expression levels between the low *C4ORF19* group and high *C4ORF19* group. **(I)** Correlation of *C4ORF19* with CD274, PDCD1, CTLA4, and HAVCR2. *means p<0.05, * * means p<0.01, * * * means p<0.001.

**Figure 19:**
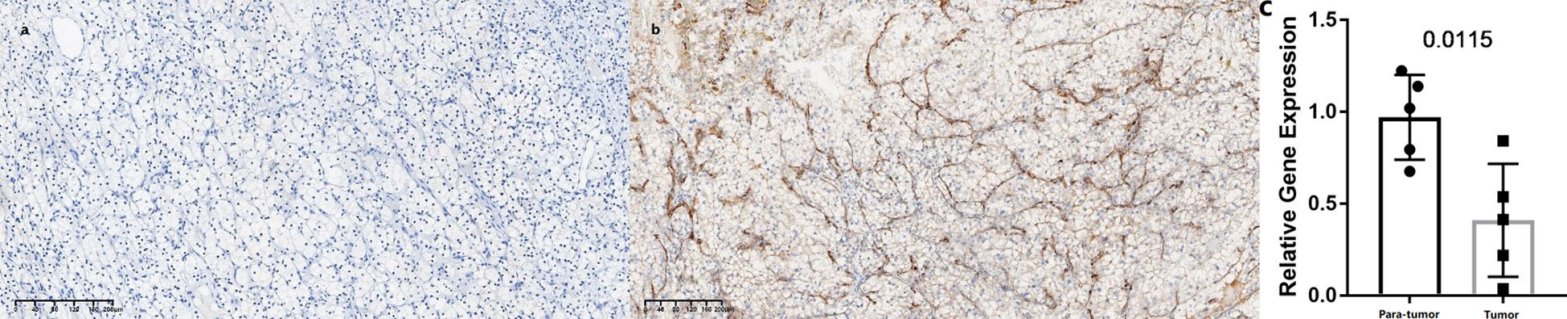
Expression levels of *C4ORF19* in ccRCC tissues. **(A)** Representative microphotographs revealed *C4ORF19* expression in tumor tissues using IHC staining. Brown, *C4ORF19*. Blue, hematoxylin. Bar = 200 μm. **(B)** Representative microphotographs revealed *C4ORF19* expression in paratumor tissues using IHC staining. Brown, *C4ORF19*. Blue, hematoxylin. Bar = 200 μm. **(C)** qPCR showed low expression of *C4ORF19* in ccRCC tissue.

In this study, we found only one prognostic AS-related gene, *C4ORF19*, whose expression level was significantly downregulated. According to the TCGA database, the expression level of *C4ORF19* in normal adjacent tissues was higher than that in tumor tissues (**Figure 18A**). The IHC experiment showed that the expression level of *C4ORF19* in ccRCC tissue was significantly lower than that in normal tissue, and the experimental results of qPCR also confirmed this point (*p* = 0.0115) (**Figure 19**). It could be clearly seen that the expression levels of *C4ORF19* in different tumor grades (**Figure 18C**, almost *p* < 0.05), different pathological stages (**Figure 18D**, almost *p* < 0.05), T state, M state, and gender (**Figures 18E–G**, almost *p* < 0.05) had significant statistical significance. In order to further assess the prognostic value of *C4ORF19* in ccRCC, K–M analyses were performed between patients with low and high expression of *C4ORF19*. A higher *C4ORF19* expression level significantly correlated with a longer overall survival time, as illustrated in **Figure 18B** (*p* < 0.001). Moreover, in 28 of 47 immune check blockade-associated genes (i.e., PDCD1, CTLA4, etc.), there were significant dysregulations in the expression levels between the low *C4ORF19* group and high *C4ORF19* group in different subgroups (**Figure 18H**). Then, a possible role for *C4ORF19* in ICB treatment of ccRCC was explored by analyzing the association between *C4ORF19* and ICB key targets adjusted for tumor purity using TIMER. The TIMER results exhibited that *C4ORF19* had a significant positive correlation with CD274 (*r* = 0.361; *p* = 1.21e−15) and HAVCR2 (*r* = 0.137; *p* = 3.15e−03) and a significant negative correlation with PDCD1 (*r* = −0.129; *p* = 5.46e−03) and CTLA4 (*r* = −0.095; *p* = 4.11e−02; **Figure 18I**), suggesting that *C4ORF19* may play a vital role in the ICB treatment of ccRCC.

### 3.8 *C4ORF19* in TIME

**Figure 20:**
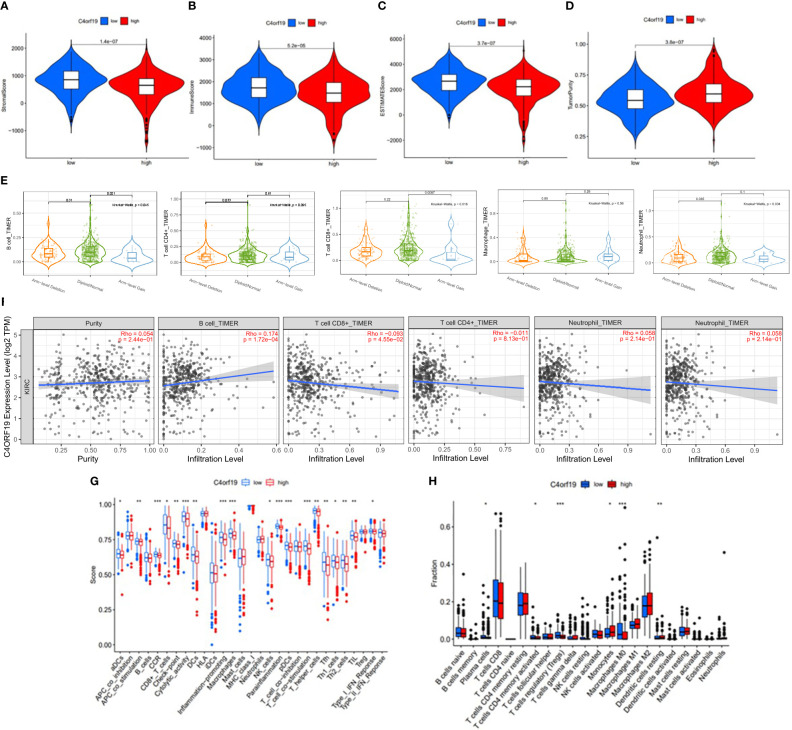
The role of *C4ORF19* in TIME features. **(A)** Comparison of stromal score between the low/high *C4ORF19* groups. **(B)** Comparison of immune score between the low/high *C4ORF19* groups. **(C)** Comparison of ESTIMATE score between the low/high *C4ORF19* groups. ESTIMATE score. **(D)** Comparison of tumor purity between the low/high *C4ORF19* groups. **(E)** Copy number of immune cells in ccRCC. **(F)** Relationship between *C4ORF19* expression level with B cells, CD8+ T cells, CD4+ T cells, macrophages, and neutrophils. **(G)** Comparison of ssGSEA enrichment between the low/high *C4ORF19* groups. **(H)** Comparison of CIBERSORT results between the low/high *C4ORF19* groups. *means p<0.05, * * means p<0.01, * * * means p<0.001.

Firstly, we classified ccRCC patients into high/low *C4ORF19* groups for further study according to the median *C4ORF19* expression level. The ESTIMATE results showed significantly higher stromal and immune scores in the low *C4ORF19* group than in the high *C4ORF19* group, suggesting more infiltration of stromal and immune cells and lower tumor purity in the low *C4ORF19* group (**Figures 20A–D**). Moreover, the relationship between the gene copy number of the different mutation types and main immune cells is exhibited in **Figure 20E**. Afterward, a positive correlation was found between *C4ORF19* expression level and B-cell infiltration, while a negative correlation was found between *C4ORF19* expression level and CD8+ T-cell infiltration. There was no significant difference in the expression level of *C4ORF19* when CD4+ T cells, macrophages, and neutrophils were infiltrated (**Figure 20F**). The consequences of ssGSEA presented that the infiltration fraction of aDCs, CD8+ T cells, DCs, macrophages, pDCs, Th1 cells, Th2 cells, NK cells, parainflammation, T helper cells, Tfh, TIL, APC co-stimulation, checkpoint, T-cell co-stimulation, CCR, cytolytic activity, inflammation-promoting, and IFN-response type-I were significantly increased when the *C4ORF19* expression level was declining (**Figure 20G**). The consequences of the CIBERSORT analysis of the TCGA cohort presented that the proportions of plasma cells, Tregs, activated memory CD4 T cells, and M0 macrophages were significantly higher and the proportions of monocytes and resting dendritic cells were significantly lower in patients with low *C4ORF19* expression (**Figure 20H**).

### 3.9 Establishment of the SF-AS regulatory network

**Figure 21:**
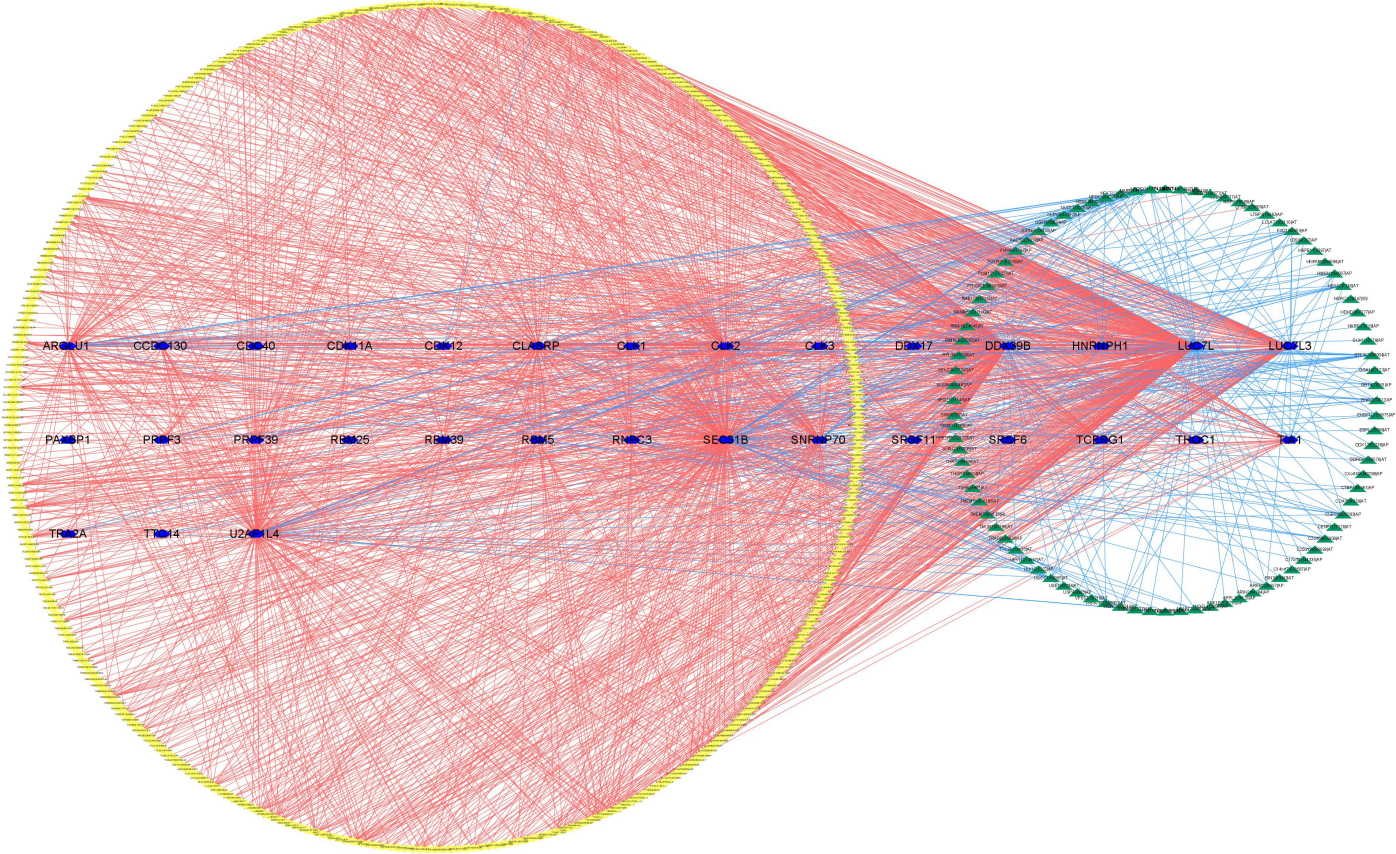
The regulatory network between SFs and survival-related AS events. The yellow or green nodes indicated that the AS events were positively or negatively correlated with survival. Blue hexagons symbolized SFs. The positive/negative correlations (*r* > 0.6 or *r* ≤ 0.6) between SFs and AS events were shown with red/blue lines.

The upregulated and downregulated genes were the results of the correlation analysis with the corresponding gene expression levels in tumor samples (**Additional file 1: Table 4**). Finally, to better explain the underlying mechanisms of AS regulation, we used 351 upregulated AS events (yellow diamond), 88 downregulated AS events (green triangle), and 31 SFs (blue hexagon; **Figure 21**) to establish the correlation network between SF expression level and PSI value of prognostic AS events. In the regulation network, the most important four nodes (**Additional file 1: Table 4**) consisting of two upregulated AS events (METTL3|26596|RI and FADS3|16305|RI) and two SFs (DDX39B and LUC7L) were identified. As a result, these SFs had great potential to further mediate the occurrence and development of tumors in ccRCC as key regulatory factors involved in abnormal AS regulation.

## 4 Discussion

Among urinary cancers, the mortality rate of renal cell carcinoma ranks first in the world (2). In addition to being one of the most malignant urologic tumors, ccRCC is also one of the most common subtypes of renal cell carcinoma (3). Genetic, molecular, and clinicopathological characteristics of ccRCC could not accurately forecast clinical therapy outcomes and the prognosis of patients (23). RCC has dissimilar immunological features in pathogenesis and treatment. Thus, there is a great need to further investigate powerful prognostic tools to predict immunotherapeutic outcomes and to recognize patients for whom immunotherapy might be effective.

Growing studies have proven that AS, which refers to a post-transcriptional modification procedure, functions in physiological and pathological processes (8). The irregular regulation of AS generally indicated that tumors occurred and developed, including ccRCC (10). Therefore, dysregulated expressed genes have the potential to be utilized as new prognostic indicators and effective therapeutic targets. Unfortunately, we still lacked enough understanding of the relationship of the AS prognostic signature with TIME and immunotherapy results in ccRCC.

In this study, we made full use of univariate Cox regression analysis. As a result, we found 9,347 AS events to be significantly associated with survival, in order to further explore the prognostic value of AS events. Afterward, based on a comprehensive bioinformatics analysis, we summarized and validated eight (AP, AD, AA, AT, ME, RI, ES, ALL) prognostic predictive signatures, all of which showed strong predictive abilities in ccRCC. In addition, when ccRCC patients were grouped according to clinicopathological stage and tumor grade, these signatures still had excellent predictive ability. We drew a nomogram to better serve the clinic. As expected, the predicted results of the nomogram were in good agreement with the actual results. As mentioned above, we developed and presented the SF-AS regulatory network to interpret the underlying mechanisms of AS regulation.

Although our new nomogram shows good predictive ability, we believe that the nomogram we created using risk score and stage cannot replace the IMDC score model and the nomograms based on clinical data at present (24, 25). This is because we did not classify renal clear cell carcinoma into metastatic and non-metastatic types, making it difficult to make accurate comparisons. Therefore, external validation of big data may be a more acceptable method to assess the effectiveness of our nomogram. However, this does not mean that our new nomogram is an invalid effort. Our proposed risk score has the potential to be an independent factor in predicting the prognosis of renal clear cell carcinoma.

By exploring the role of AS events in TIME with the method described above, we found that there was generally a high level of infiltration and a more active immune state in the high-risk group, which indicates that immune recognition and antitumor effects are present. Moreover, these results suggested that risk scores could facilitate the prediction of immunotherapy outcomes. Unfortunately, we had no way to explore the association between risk score and ICB treatment outcomes because there was no ICB treatment dataset in the ccRCC cohort. Then, risk score had a significantly positive relationship with PDCD1 and CTLA4 and a significantly negative relationship with HAVCR2 and IDO1. Furthermore, it was worth mentioning that risk score was significantly connected with 36 (i.e., HHLA2, etc.) ICB gene expression levels. These results above confirmed that risk scores did have the potential to help develop more scientific and personalized immunotherapy strategies.

*C4ORF19* (Chromosome 4 Open Reading Frame 19) is a protein-coding gene. Wang W. et al. reported that regulated *C4ORF19* could promote colon adenocarcinoma cell proliferation, invasion, and migration (26). However, our understanding of the role of *C4ORF19* in clear cell renal carcinoma is not clear so far. This study indicated that *C4ORF19* was significantly downregulated in cell lines, largely suggesting a poor prognosis for ccRCC. In ICB immunotherapy for ccRCC, the *C4ORF19* expression level correlated significantly with clinicopathological stage, tumor grade, and key genes (i.e., IDO1). However, the potential biological role of *C4ORF19* was unclear and required further study.

In general, ccRCC patients with higher risk scores or lower levels of *C4ORF19* expression had higher levels of immune cell infiltration in the tumor environment, suggesting immunophenotypic activation, but shorter overall survival. Therefore, we hypothesized that the ICB pathways might influence the antitumor effect of immune cells, and the risk score was related to the expression of immune checkpoint blockade targets.

This study had the following advantages in exploring new prognostic factors for ccRCC. First of all, as a result of this study, we were able to uncover the role of AS events in the formation of TIME diversity and complexity as well as their role in the prediction of ICB therapy outcomes, which had not been clarified. In addition, to uncover the comprehensive landscape of TIME in ccRCC, the ESTIMATE R package, ssGSEA algorithm, CIBERSORT method, and TIMER database exploration were employed. Finally, the study emphasized the biological function of *C4ORF19* in clear cell renal carcinoma for the first time.

The current research also had several shortcomings. First of all, the AS events in ccRCC were investigated using the public TCGA cohort, which was not validated using the in-house cohort. In addition, the effectiveness of prognostic indicators including the ALL prognostic signature and prognostic nomogram still needed to be verified through clinical trials. Furthermore, the conclusion that the key gene *C4ORF19* was downregulated in ccRCC tumor tissue still required a larger number of experiments.

## 5 Conclusion

All in all, we systematically analyzed the prognostic value of RNA splicing patterns in order to strengthen the prognostic prediction of ccRCC. The nomogram we developed using risk score and stage is not as effective in predicting prognosis compared with the nomogram based on clinical data. Despite this, our proposed risk score has the potential to be an independent factor in predicting the prognosis of ccRCC. In addition, the promising targets for ccRCC antitumor therapy were identified from the AS-SF regulatory network. After comprehensive bioinformatics analysis of AS events, the AS atlas was closely correlated with the TIME characteristics and immunotherapy of ccRCC. However, these findings still required more experimental and clinical exploration to verify. At the same time, the mechanism of tumor occurrence and development of ccRCC and the impact of these AS events still need to be further explored.

## Data availability statement

The datasets presented in this study can be found in online repositories. The names of the repository/repositories and accession number(s) can be found in the article/**Supplementary Material**.

## Ethics statement

The studies involving humans were approved by the Hospital Ethics Committees of the second Affiliated Hospital of Wenzhou Medical University. The studies were conducted in accordance with the local legislation and institutional requirements. The participants provided their written informed consent to participate in this study.

## Author contributions

JZ mainly completes bioinformatics analysis and article writing; HJ mainly completes experimental verification; As the first corresponding author, XJ guides the whole process; As the second corresponding author, DR provides experimental help. All authors contributed to the article and approved the submitted version.
